# 

**Published:** 1859-07

**Authors:** 


VOL. II.] PUBLISHED MONTHLY. [No. 7.

### THE CHICAGO MEDICAL JOURNAL.

EDITED BY

DANIEL BRAINARD, M. D.

Professor of Surgery in Rush Medical College; Surgeon to the United States Marine Hospital, etc.

ASSISTED BY

EDWIN POWELL, M. D.

DEMONSTRATOR OF ANATOMY IN RUSH MEDICAL COLLEGE.

[v.16]

JULY, 1859.

CHICAGO:

JAMES BARNET, BOOK AND JOB PRINTER, 189 LAKE STREET.

AT TWO DOLLARS PER ANNUM, IN ADVANCE.

